# Associations between primary care practice type and patient-reported access

**DOI:** 10.1186/s12913-018-3590-z

**Published:** 2018-10-16

**Authors:** K Selby, J-C Zuchuat, C Cohidon, N Senn

**Affiliations:** 1 Department of Ambulatory Care and Community Medicine, University of Lausanne, Lausanne, Switzerland; 2 Bureau d’étude JC Zuchuat, 1806, St-Légier, Switzerland; 3 Institute of Family Medicine, Department of ambulatory care and community medicine, University of Lausanne, Lausanne, Switzerland

**Keywords:** Primary care, Comprehensiveness, Access, Switzerland

## Abstract

**Background:**

We recently defined a global typology of primary care (PC) in Switzerland using a mixed inductive/deductive approach to construct latent, composite variables that summarize variance between practices. Now we explore associations between the primary variable that describes the comprehensiveness of services and patient-perceived access to PC in Switzerland.

**Methods:**

Cross-sectional surveys were administered to physicians from the Swiss PC Active Monitoring (SPAM) network and their patients. The primary outcomes were patient responses to: “Was it easy to get the appointment?”, “The opening hours are too restricted” and “In the past 12 months, did you postpone or abstain from a visit to this doctor or another GP when you needed one?” Multivariate, multilevel analyses with stepwise regression were used to assess associations between practice type (practices with a broader range of services have higher scores) and perceived access, controlling for patient characteristics.

**Results:**

One hundred and ninety nine of 200 PC physicians in the network completed the questionnaire. Of 2628 patients approached after a physician visit, 1791 accepted (participation = 76%), with 9 patients at each practice. No association was observed between comprehensiveness of services and difficulty getting an appointment. When controlling for patient factors, there was a weak association between higher scores for comprehensiveness of services and patients reporting that the opening hours are too restricted (*p* = 0.05), though this was no longer significant after controlling for language area. Greater comprehensiveness of services was associated with fewer patients needing to postpone visits (OR 0.93, 95%CI 0.88–0.99, *p* = 0.03).

**Conclusions:**

Though fewer patients report needing to postpone visits at practices with more comprehensive offering of services, there is limited evidence of associations between patient-reported access and a global typology of Swiss primary care.

## Background

Adequate, timely access to high-quality care is a cornerstone of primary care (PC) [1]. PC is meant to be where people have their “first contact care, health promotion and basic treatment”, and their PC physician (PCP) is meant to facilitate “adequate access to other health care and related services for those who need this” [1]. Decreased access to PC has been associated with increased use of more expensive services such as the emergency room or medical specialists [2], while improved access to PC may increase the use of preventive services [3]. Access to PC is a complex concept that involves multiple health system, practice and patient factors [4]. Previous research of patient factors has found that younger patients and those from ethnic minorities report worse access [5–7]. Access is variably measured by patient reported outcomes measures or by attempts to find more ‘objective’ measures such as the next available appointment, but no consensus appears to exist [8].

Traditional determinants of PC access at the practice-level have frequently been identified using practice size and reimbursement models for physicians [9, 10]. Associations between PC practices grouped by organization or services offered and access are particularly important to guide organizational changes in primary care to improve access. Health systems can also use incentives to encourage certain practice types associated with improved patient access [11]. From previous literature, patients seem to report easier access to smaller practices [7, 9, 12], and access and continuity may be in opposition to each other, where practices with improved access have worse continuity [9, 10]. One limitation of this previous literature has been difficulty integrating the many aspects of PC practice organization into analyses of the determinants of access. Typically, a small number of predefined PC characteristics are used as independent variables to assess patient access, leading to only a partial view.

Our team recently employed a novel methodology to create a typology of PC practices that integrates many variables simultaneously using a multiple factorial analysis [13]. We inputted 74 PC features from the QUALICOPC questionnaire integrating multiple organizational features of PC practices that were then summarized with two axes, thus creating an empirical typology. We found this approach helpful for describing Swiss PC as it is made up of a heterogeneous mix of general practitioners and general internists with the liberty to define their scope of practice [14]. Pre-defined models commonly used in other countries (like for example community health centres), do not readily apply.

The first of the two composite axes contained variables related to the range of clinical and technical procedures provided, and was named “comprehensiveness of services” score [13], while the second axis contained variables related to the workforce available in each practice. The comprehensiveness of services axis is of particular interest as there appears to be a gradual narrowing of the PCP scope of practice in Switzerland [15].

Using the defined latent variables, the current study aimed to investigate the relationship between access and PC practice type, using data from a large, practice-based network of PCPs and their patients in Switzerland. We hypothesized that PC practice type, as characterized by a practice’s score on the comprehensiveness axis, would be a predictor of patient-perceived access.

## Methods

### Context and setting

Data for this analysis came from PCP and patient responses to the Quality and Costs of Primary Care in Europe (QUALICOPC) survey, collected in Switzerland between January and June 2012. QUALICOPC was a multinational survey funded by the European Commission to compile data from 31 European countries and 3 non-European countries with standardized questionnaires [16]. Each country’s primary care system was evaluated against criteria of quality, equity and cost. The Department of Ambulatory Care and Community Medicine of the University of Lausanne was chosen to coordinate data collection for Switzerland. Data collection relied on cross-sectional surveys of PCPs in the Swiss Primary Care Active Monitoring (SPAM) network and their patients. The SPAM network was formed by inviting a random sample of PCPs stratified by area so as to have a nationally representative sample of PCPs [17]. The SPAM network appears to be representative of Swiss PCPs based on several demographic variables [17]. Swiss primary care is provided by a mix of specialists in general medicine and general internal medicine, though the two professional organizations have recently merged for a common specialization [14].

### Data collection and questionnaire

In each PCP’s practice of the SPAM network, trained field workers invited consecutive patients 18 years or older after they had had a face-to-face consultation with the PCP to participate in the study until 9 patients had completed a Patient Experiences questionnaire (used for this analysis) and 1 a Patient Values questionnaire [18]. PCP’s also completed a questionnaire related to the organization of their practice and what clinical activities they frequently perform, which were linked to the responses of his or her patients, allowing for multi-level analyses of the data. Local approval for this study was obtained from the ethical review board of the University of Lausanne.

The methodology used to develop the PCP and the patient experience questionnaires has been detailed elsewhere [19]. The questionnaires were translated into German, French and Italian (three national languages of Switzerland). The PCP questionnaire contained 60 questions including demographic characteristics, contextual features of their practice structure and organization, and services that they offered. The patient experience questionnaire contained 41 questions related to demographic characteristics, access to care, relationship with the doctor they saw, comprehensiveness of care offered, care coordination and use of healthcare services.

### Primary care practice type variable (comprehensiveness axis)

In order to summarize the characteristics of each practice, previous research generated two composite axes using an iterative multiple factor analysis on 74 organizational features collected through the PCP questionnaire from the QUALICOPC survey [13]. From the ~ 150 practice-level variables available, half were excluded because they were irrelevant in Switzerland (ex: capitation does not exist), or because of very low variance. A hierarchical method was then used to give equal weight to available variables in 5 groups: practice infrastructure, clinical care, workforce distribution, accessibility and geographical location. The two axes represent the main orientation of the PCP practices in this sample, and are referred to as composite scores for the comprehensiveness of services and for practice workforce. They represent the main global features to characterize practice models in our Swiss representative sample. They vary from − 10 to 10, centered on the centroid, though their precise numerical scores do not have a direct meaning.

The comprehensiveness axis explains 11% of the variance seen in the PCP practice dataset [13] and contains variables related to infrastructures and clinical care. PCP questions related to higher comprehensiveness scores were: for clinical care, reporting that patients come to them first for a broad range of somatic complaints and procedures, such as acute illness in children and small surgical procedures (ex: wound suturing, cyst removal), but less for adult psychosocial complaints such as anxiety; and for infrastructures, PCPs reporting that they have a broad range of technical, laboratory, and radiology equipment in their practice rather than available nearby (ex: set for minor surgery and basic x-ray).

The practice workforce axis explains 6% of the variance and contains variables related to workforce distribution. The practice workforce score was excluded from our statistical models presented in this paper because it was unstable at higher values due to the small number of large practices in Switzerland.

### Access variables

For this analysis, we focused on three primary outcomes. Primary outcomes were the responses to the questions “Was it easy to get this appointment”, “The opening hours are too restricted” and “In the past 12 months, did you postpone or abstain from a visit when you needed one?”. The “easy to get an appointment” question was derived from the Primary Care Assessment Tool [20], which has been used extensively in other studies [21]. The “opening hours” question was taken from a Commonwealth Fund questionnaire for adults with chronic conditions [22]. The “postpone” question was newly developed for QUALICOPC [18]. Other access related questions were not retained to prioritize questions directly describing access and most relevant to Switzerland. For example, distance needed to travel is primarily important in sparsely populated rural areas, rare in Switzerland, and several questions, such as whether someone needed to wait when calling the office, had too few positive answers to allow for meaningful comparisons.

### Statistical procedures

Descriptive statistics, with frequencies and means, were used to present patient and physician characteristics. We built logistic regression models for the three primary outcomes. Given the nested nature of the patient results, we performed multilevel analyses with random intercept, controlling for clustering at the level of individual practices. First, for each of the three outcomes, as dependent variables, univariate regressions were performed to study their association with the practice’s composite score for the comprehensiveness of services and various patient and practice’ variables, as independent variables. Variables significant to a *p*-value < 0.2 were retained for a multivariate model. Due to a very important collinearity between the score of comprehensiveness and the linguistic area and to avoid overadjustment, we built two final models, with and without adjustment on the linguistic area. Stepwise regression was used to identify the strongest variables, given the large number of potentially correlated patient variables, e.g. patient level of education and income were not studied simultaneously in a same model. Given the very small number of missing responses, patients with missing data were excluded from regression models and assumed to be missing at random. Analyses were performed using R version 3.2.2, the lme4 package version 1.1–9 and the MuMIn package version 1.15.1.

## Results

Of the 200 PCPs of the SPAM network, 199 completed the questionnaire. Characteristics of the 1791 patients recruited after a visit in each of these practices are shown in Table 1. The mean age was 57 years, 56% were female, 45% reported having a longstanding disease or condition, and 33% described their own health as fair or poor. The majority (91%) had a visit with their usual PCP, and the most common reasons for a visit were illness (37%) and a check-up (33%).

**Table 1:** Patient and visit characteristics (*n* = 1791)

Patient demographics	
Sex – female	1009 (56%)
Age, years	57 (±0.44)
Currently employed	619 (35%)
Retired	749 (42%)
Self-perceived as average income compared for region	1312 (73%)
Completed post-secondary education	305 (17%)
Born in Switzerland	1331 (74%)
Speaks the local official language fluently	1417 (79%)
Patient health-related characteristics	
How would you describe your own health? (Fair or poor)	590 (33%)
Do you have a longstanding disease or condition? (yes)	800 (45%)
Do you have your own doctor? (yes, the one I visited)	1629 (91%)
Do you have your own doctor? (yes, another one)	100 (6%)
Visit characteristics	
What is the reason for your visit?	
Illness	670 (37%)
Check-up	590 (33%)
Other	530 (30%)
Physician Demographics	
Sex (female)	44 (22%)
Age, years	55 (±0.57)
Employment status	
Salaried (%)	9 (4.5%)
Self-employed (%)	194 (97%)
Practice characteristics	
Comprehensiveness score	55 (±0.57)
Linguistic area	
German speaking	123 (62%)
French speaking	69 (35%)
Italian speaking	8 (4%)
Practice location	
City / suburbs	61 (31%)
Small town	34 (17%)
Mixed urban / rural	50 (25%)
Rural	52 (26%)

The overall access results are shown in Table 2. Ninety-two percent of patients had made an appointment for their visit, though 42% had made the appointment the same day or the day before. Of those who had made an appointment, 31% responded that it was not easy to get the appointment. Few patients reported having had to miss or postpone a visit in the past 12 months (10%), and only 5% because of lack of insurance or other financial reasons.

**Table 2:** Patient perception of their access to primary care (*n* = 1791)

Did you make an appointment for this visit? (yes)	1649 (92%)
Was it easy to get the appointment?	
Yes	1089 (61%)
No	549 (31%)
I don’t know	134 (7%)
How many days did you wait for this visit?	
Same day or waited 1 day	696 (42%)
Waited 2–7 days	448 (27%)
Waited more than a week	169 (10%)
I don’t know	278 (17%)
Do you agree that the opening hours are too restricted?	
Yes	142 (8%)
No	1524 (85%)
I don’t know	97 (5%)
In the past 12 months, did you postpone or abstain from a visit to this doctor or another general practitioner when you needed one?	
Yes	170 (10%)
Most important reason for postponing:	
I did not have insurance	1 (1%)
Other financial reasons	6 (4%)
I could not get there (physically)	20 (12%)
I was too busy	39 (24%)
Other reason	95 (59%)
No	1610 (90%)

In univariate analyses, few associations were observed between the difficulty to obtain an appointment and patient- and practice-level variables, except for a higher level of education and the French linguistic area (Table 3). However, education level and language area did not remain statistically significant in the multivariate final model (Table 3).

**Table 3:** Logistic regression results for difficulty getting an appointment (*n* = 1649)

Variable	Univariate analyses		Multivariate final models			
			Without linguistic area		Including linguistic area	
	Odds Ratio (95% CI)	*p*-value	Odds Ratio (95% CI)	*p*-value	Odds Ratio (95% CI)	*p*-value
Practice comprehensiveness of services score						
Increased score	1.10 (0.95–1.27)	0.20	1.10 (0.95–1.27)	0.18	1.00 (0.86–1.18)	0.96
Patient sex						
Male	1.39 (0.67–2.86)	0.38				
Patient age						
Increased age (years)	1.00 (0.99–1.03)	0.48				
Patient born in Switzerland						
Born outside Switzerland	1.51 (0.71–3.24)	0.29				
Patient employed or retired						
Unemployed or problem working	0.41 (0.05–3.10)	0.38				
Patient has not completed secondary education						
Secondary	0.59 (0.26–1.37)	0.22	0.57 (0.25–1.31)	0.18	0.57 (0.25–1.31)	0.19
Post-secondary or more	**0.34 (0.12–1.00)**	**0.05**	**0.34 (0.11–0.98)**	**0.05**	0.38 (0.13–1.10)	0.08
Patient suffers from a chronic disease						
No	0.67 (0.33–1.37)	0.27				
Speaks the official language fluently						
Sufficiently well	0.71 (0.15–3.34)	0.67				
Moderately well	0.96 (0.15–6.06)	0.97				
Poorly	–	–				
Not at all	–	–				
Income lower than country average						
Average	0.82 (0.29–2.31)	0.71				
Above average	0.43 (1.00–1.95)	0.28				
General health status is very good						
Good	1.15 (0.44–3.04)	0.78				
Fair	0.79 (0.25–2.50)	0.69				
Poor	–	–				
Reason for visit was acute illness						
Other reason	**0.58 (0.28–1.17)**	**0.13**				
Visit was with patient’s own doctor						
Not with patient own doctor	**2.15 (0.78–5.85)**	**0.14**				
Linguistic area -German speaking						
French speaking	**0.27 (0.08–0.85)**	**0.03**			2.28 (0.08–1.07)	0.06
Italian speaking	0.36 (0.07–1.82	0.22			0.442 (0.09–2.26)	0.33
Urban area						
Rural area	1.06 (0.44–2.56)	0.89				

**Bold:** significant to < 0.2 in univariate analyses or < 0.05 in multivariate analyses

For the second primary outcome (“opening hours too restricted”), the univariate regressions were significant for several patient characteristics, such as level of education, income and linguistic area. Moreover, higher practice comprehensiveness scores were moderately associated with more patients reporting that the opening hours are too restricted (OR 1.07, 95% CI 1.00–1.14, *p* = 0.04). This association remained in the multivariate regression OR 1.07, 95% CI 1.00–1.14, *p* = 0.05), but totally disappears when the variable linguistic area is included in the final model (Table 4).

**Table 4:** Logistic regression results for question, “The visiting hours are too restricted” (*n* = 1791)

Variable	Univariate analyses		Multivariate final models			
			Without linguistic area		Including linguistic area	
	Odds Ratio (95% CI)	*p*-value	Odds Ratio (95% CI)	*p*-value	Odds Ratio (95% CI)	*p*-value
Practice comprehensiveness of services score						
Increased score	**1.07 (1.00–1.14)**	**0.04**	**1.07 (1.00–1.14)**	**0.05**	1.00 (0.93–1.07)	0.89
Patient sex (female)						
Male	0.78 (0.55–1.16)	0.24				
Patient age						
Increased age (years)	**1.01 (1.00–1.02)**	**0.16**	1.17 (0.98–1.41)	0.09	1.18(0.99–1.42)	0.07
Patient born in Switzerland						
Patient born outside of Switzerland	0.93 (0.61–1.43)	0.74				
Patient employed or retired						
Unemployed or problem working	0.95 (0.48–1.89)	0.89				
Patient has not completed secondary education						
Secondary	**1.72 (0.96–3.09)**	**0.07**	**1.72 (0.95–3.09)**	**0.07**	1.71 (0.95–3.07)	0.07
Post-secondary or more	**1.87 (1.00–3.46)**	**0.05**	**1.98 (1.06–3.68)**	**0.03**	**2.11 (1.13–3.92)**	**0.02**
Patient suffers from a chronic disease						
No	1.04 (0.72–1.51)	0.82				
Speaks the official language fluently						
Sufficiently well	0.99 (0.55–1.19)	0.97				
Moderately well	**1.78 (0.91–3.44)**	**0.09**				
Poorly	0.67 (0.09–5.17)	0.70				
Not at all	2.55 (0.52–12.58)	0.25				
Income lower than country average						
Average	**1.63 (0.80–3.33)**	**0.18**				
Above average	**2.29 (1.03–5.08)**	**0.04**				
General health status is very good						
Good	1.11 (0.64–1.95)	0.70				
Fair	**1.58 (0.87–2.87)**	**0.13**				
Poor	1.58 (0.70–3.57)	0.27				
Reason for visit was acute illness						
Other reason	0.89 (0.60–1.26)	0.46				
Visit was with patient’s own doctor						
Not with patient own doctor	0.95 (0.47–1.90)	0.88				
Linguistic area -German speaking						
French speaking	**0.46 (0.29–0.73)**	**< 0.001**			**0.43 (0.25–0.74)**	**< 0.01**
Italian speaking	**0.23 (0.09–0.59)**	**0.02**			**0.23 (0.09–0.60)**	**< 0.01**
Urban area						
Rural area	1.01 (0.68–1.50)	0.97				

**Bold:** significant to < 0.2 in univariate analyses or < 0.05 in multivariate analyses

Regarding the last outcome, there was an association between increasing comprehensiveness of services offered and patients reporting that they had to postpone their visit in univariate analysis (OR 0.91, 95% CI 0.86–0.96, *p* = 0.001) and in the multivariate final model (OR 0.92, 95% CI 0.87–0.97, *p* = 0.002), even after including the linguistic area variable (Table 5). The other predictive variables were patients’ age (OR 1.38, 95% CI 1.15–1.65, *p* < 0.001), the number of visits during the last 6 months (OR 1.23, 95% CI 1.03–1.46, *p* = 0.02) and a fair or poor perceived health (OR 2.28, 95% CI 1.06–4.91, *p* = 0.03).

**Table 5:** Logistic regression results for question “In the past 12 months, did you postpone or abstain from a visit when you needed one?” (*n* = 1791)

Variable	Univariate analyses		Multivariate final models			
			Without linguistic area		Including linguistic area	
	Odds Ratio (95% CI)	*p*-value	Odds Ratio (95% CI)	*p*-value	Odds Ratio (95% CI)	*p*-value
Practice comprehensiveness of services score						
Increased score	**0.91 (0.70–0.96)**	**0.001**	**0.92 (0.87–0.97)**	**< 10** ^**−2**^	**0.93 (0.88–0.99)**	**0.03**
Patient sex						
Male	0.99 (0.70–1.40)	0.0.97				
Patient age						
Increased age (years)	**1.01 (1.00–1.02)**	**0.01**	1.38 (1.15–1.65)	**< 0.001**	1.40(1.17–1.68)	**< 0.001**
Patient born in Switzerland						
Born outside Switzerland	**1.58 (1.09–2.27)**	**0.02**				
Patient employed or retired						
Unemployed or problem working	**2.08 (1.26–3.43)**	**0.04**				
Patient has not completed secondary education						
Secondary	0.86 (0.53–1.39)	0.54				
Post-secondary or more	1.23 (0.75–2.03)	0.41				
Patient suffers from a chronic disease						
No	0.90 (0.64–1.27)	0.55				
Speaks the official language fluently						
Sufficiently well	0.90 (0.51–1.58)	0.71				
Moderately well	1.34 (0.68–2.65)	0.40				
Poorly	–	–				
Not at all	1.89 (0.38–9.39)	0.44				
Income lower than country average						
Average	0.93 (0.54–1.61)	0.81				
Above average	1.43 (0.76–2.71)	0.27				
General health status is very good						
Good	1.10 (0.63–1.92)	0.73	1.19 (0.67–2.10)	0.55	1.11 (0.63–1.95)	0.72
Fair	**1.99 (1.12–3.52)**	**0.02**	**2.04 (1.11–3.77)**	**0.02**	**1.84 (1.00–3.40)**	**0.05**
Poor	**2.41 (1.18–4.97)**	**0.02**	**2.28 (1.06–4.91)**	**0.03**	**2.32 (1.08–4.97)**	**0.03**
Reason for visit was acute illness						
Other reason	0.95 (0.67–1.36)	0.80				
Visit was with patient’s own doctor						
Not with patient own doctor	1.37 (0.76–2.45)	0.29				
Number of visits in the last 6 months						
	**1.29 (1.09–1.52)**	**< 0.01**	**1.23 (1.03–1.46)**	**0.02**	**1.23 (1.06–1.51)**	**0.01**
Linguistic area -German speaking						
French speaking	**1.79 (1.20–2.65)**	**0.01**			**1.23 (0.76–1.97)**	**0.01**
Italian speaking	**3.67 (2.29–5.89)**	**< 0.001**			**3.39 (2.10–5.49)**	**< 0.01**
Urban area						
Rural area	**0.55 (0.38–0.81)**	**0.01**				

**Bold:** significant to < 0.2 in univariate analyses or < 0.05 in multivariate analyses

## Discussion

In this cross-sectional, nationally representative survey of Swiss PCPs and their patients, we found limited evidence of associations between a novel typology of PC practices defined by the comprehensiveness of services offered and patient-reported access. There was a slight trend of patients seen at offices with more comprehensive services being less likely to have postponed an appointment within the last 12 months.

It is unclear why organizational differences between PC practices, as described by the comprehensiveness axis from the new typology, would have a limited impact on patient-reported access in Switzerland. One possibility is that patient access is generally quite good in Switzerland, creating a ceiling effect that makes it difficult to differentiate between practices. The fact that we only interviewed patients after an appointment, who by definition got access to a PCP, may add to that effect. A 2014 telephone survey of older adults conducted by the Commonwealth Fund found that Switzerland did quite well on overall indicators of access to primary care [23]. However, direct comparisons with Canada and Greece, who also participated in QUALICOPC, show that a higher proportion of patients in both say it was easy to make an appointment (94% in Canada, 86% in Greece and 61% in the current study) [24, 25]. Research from other countries focusing on first-contact accessibility has shown that access appears to be better in small clinics [9] and with fee-for-service [26], which are the norm in Swiss PC. American community health centres appear to do less well with first-contact accessibility than other models [27]. However, Switzerland does not perform well on all aspects of patient access, in particular with regards to weekend access [28]. It is equally possible that while the comprehensiveness access best describes variability between Swiss PC practices, other aspects of the practices not included in the comprehensiveness score are more important for patient access. Finally, it is possible that differences between practices are in fact relatively minor, as supported by our previous study showing that one single model is predominant in Switzerland [13]. More important differences in primary care access might be seen when comparing internationally, a direction of future collaboration with the IMPACT (Innovative Models Promoting Access-to-Care Transformation) program [29].

Differences between Swiss linguistic regions appear to have a strong effect on patient-reported access, which could be both due to differences between PC practices and cultural differences between patients. Many of the differences between services offered that are included in the composite score are heavily influenced by regional differences that follow linguistic lines. We presented models both with and without language area as the introduction of the language area along with the comprehensiveness score may cause over-adjustment bias. Multiple previous reports have underlined the importance of patient factors, including worse access for various groups (linguistic or racial minorities, younger patients, etc.) [5, 6, 30], and a British study finding that the primary predictor of variations in patient satisfaction was unmeasured patient factors [31]. Several predictors of access that are important in other countries did not have significant associations in our study, such as financial status or insurance [32].

It is unclear why patients at practices offering more comprehensive services were less likely to have postponed an appointment within the last twelve months. In our sample, very few patients reported having to forego care for financial reasons (Table 2). Switzerland has universal coverage with private insurance, but the highest out-of-pocket medical expenses in the world [23]. Nonetheless, previous studies support the finding that inadequate finances are rarely an issue [33]. Practices with high comprehensiveness scores are more oriented towards somatic complaints and may be more flexible in their accommodation of urgent complaints. Reasons for postponing may be more often linked to lack of time or a lack of availability on nights and weekends. This may explain why age and education level have an important impact on patient answers to the statement that “the visiting hours are too restricted.”

More comprehensive offering of services in PC in the United States was recently linked to lower overall costs, suggesting that the scope of services offered could be linked to important patient outcomes [34]. Our study found only limited associations between comprehensive services and patient access. It is important to understand the impact of practice differences on access, as in both Switzerland and the United States, PCPs report a gradual narrowing of their scope of care, decreasing the comprehensiveness of services offered in individual practices [35].

### Strengths and limitations

To the best of our knowledge, an inductive approach to defining a global practice typology has not been used previously. As patients were included following consultations with a physician during regular business hours, we did not capture those people who were unable to visit a physician because of inadequate access. That means that we can only make limited inferences to access to PC on a population level. However, over two-thirds of the Swiss population, including over 80% of those over age 65, report having seen a PCP over the last twelve months [36]. This limitation is compensated by the advantage of administering the questionnaire immediately after an appointment, providing PCPs with actionable evidence from within practices.

Other strengths of our study included a large patient sample, the direct link made between patient data and individual PCPs, and the richness of information available concerning the PCP practices.

Our measures of access are patient reported outcome measures, which may be less objective than measures taken by standardized observers. However, perceived access may incorporate more elements important to a global view of patient access than simply openings in the PCP’s agenda [8]. Our third access outcome asked patients about missing visits to “this doctor or another general practitioner” (Table 2), such that patients could be referring to another PCP, thus attenuating potential associations between practice type and access. However, given that 91% of patients saw their own PCP (Table 1), that effect should be minor. We chose a priori three QUALICOPC questions with direct relevance to patient-perceived access, but access encompasses many concepts [4]. Other access variables could be more strongly associated with practice type than those we chose. These data are also observational and cross-sectional, and as a result do not provide evidence for a causal link between greater comprehensiveness and decreases in postponing appointments. As always, there is a risk of selection bias in the PCPs and patients who chose to participate. However, patient acceptance rates were quite high (76%) and the PCPs in the SPAM research network have a similar demographic profile to the overall population of Swiss PCPs [17].

We chose to examine access alone, and not other features important for high quality PC; previous studies have suggested that patient continuity and access may be opposed to each other, and it is difficult for practices to provide both [9]. Future research could examine links between practice type and continuity to see if continuity and access are in opposition to each other.

Finally, this initial analysis included data from Switzerland alone and the comprehensiveness score has not been used in other contexts. However, the results may be applicable in other countries with similar PC landscapes, such as Germany. Efforts are underway to use this new methodology on an integrated database including multiple countries.

## Conclusions

In conclusion, there WAS limited evidence for an association between the typology of practices created by our group using a multiple factorial analysis and patient-reported access. This work is an important proof of concept that a global typology capturing variance between PC practices can be applied to patient-reported measures such as access. Future studies could employ the current methods to explore associations between practice type and access across countries.

## Abbreviations

OR: Odds ratio
PC: Primary care
PCP: Primary care physician
QUALICOPC: Quality and Costs of Primary Care in Europe
SPAM: Swiss Primary care Active Monitoring
